# Supplementary figures and images for: Multi-Omics Analysis of Glioblastoma and Glioblastoma Cell Line: Molecular Insights Into the Functional Role of GPR56 and TG2 in Mesenchymal Transition
Source: Front Oncol. 2022 May 3;12:841890. doi: 10.3389/fonc.2022.841890 (PMC9119646; doi:10.3389/fonc.2022.841890)

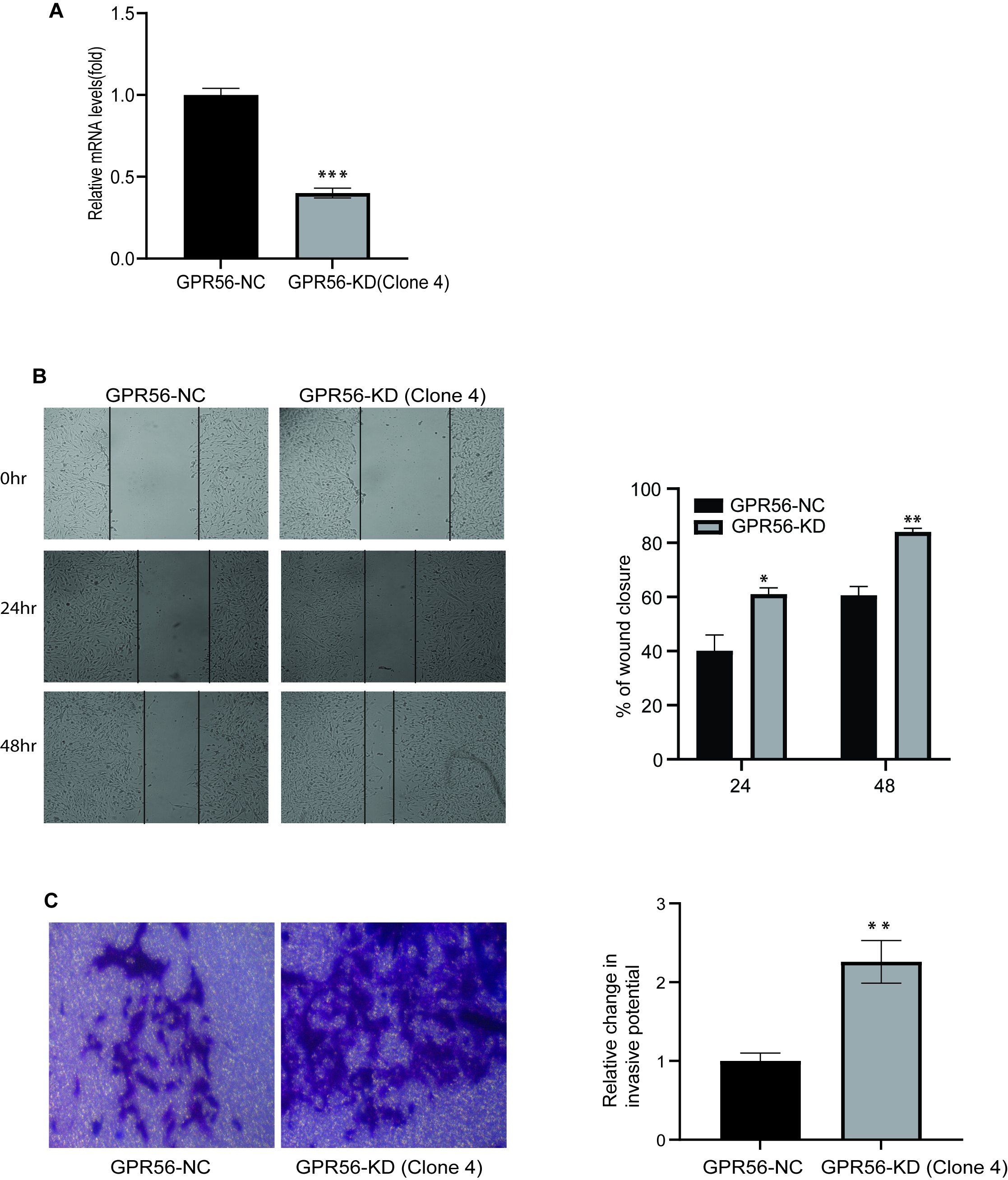

Supplement: Supplementary Figure 1 — (A) GPR56 mRNA expression analysis of GPR56-NC and GPR56-KD (clone 4) cells, error bar represents standard deviation; ***indicates p < 0.001. (B) Transwell invasion assay to determine invasive properties of GPR56-KD clone 4 cells. (C) Wound healing assay for GPR56-KD cells (clone 4) and control cells. Phase-contrast images and summary data (n=3) are given on the left and right, respectively. Details of the assays are described under methods. The wound closure percent over time was compared for the control and the knockdown cells. *indicates p < 0.05, **indicates p < 0.01. Abbreviations are GPR56-NC (GPR56 control), GPR56-KD clone 4 (GPR56 knockdown). [file Image_1.jpg]
